# Mechanical ventilation strategies for intensive care unit patients without acute lung injury or acute respiratory distress syndrome: a systematic review and network meta-analysis

**DOI:** 10.1186/s13054-016-1396-0

**Published:** 2016-07-22

**Authors:** Lei Guo, Weiwei Wang, Nana Zhao, Libo Guo, Chunjie Chi, Wei Hou, Anqi Wu, Hongshuang Tong, Yue Wang, Changsong Wang, Enyou Li

**Affiliations:** Department of Anesthesiology, The First Affiliated Hospital of Harbin Medical University, No 23 Youzheng St., Nangang District, Harbin, Heilongjiang 150001 China

**Keywords:** Ventilation strategies, ICU patients without ALI or ARDS, Network meta-analysis, Pulmonary compliance, PaO_2_/FIO_2_ ratio

## Abstract

**Background:**

It has been shown that the application of a lung-protective mechanical ventilation strategy can improve the prognosis of patients with acute lung injury (ALI) or acute respiratory distress syndrome (ARDS). However, the optimal mechanical ventilation strategy for intensive care unit (ICU) patients without ALI or ARDS is uncertain. Therefore, we performed a network meta-analysis to identify the optimal mechanical ventilation strategy for these patients.

**Methods:**

We searched the Cochrane Central Register of Controlled Trials (CENTRAL) in the Cochrane Library, EMBASE, MEDLINE, CINAHL, and Web of Science for studies published up to July 2015 in which pulmonary compliance or the partial pressure of arterial oxygen/fraction of inspired oxygen (PaO_2_/FIO_2_) ratio was assessed in ICU patients without ALI or ARDS, who received mechanical ventilation via different strategies. The data for study characteristics, methods, and outcomes were extracted. We assessed the studies for eligibility, extracted the data, pooled the data, and used a Bayesian fixed-effects model to combine direct comparisons with indirect evidence.

**Results:**

Seventeen randomized controlled trials including a total of 575 patients who received one of six ventilation strategies were included for network meta-analysis. Among ICU patients without ALI or ARDS, strategy C (lower tidal volume (VT) + higher positive end-expiratory pressure (PEEP)) resulted in the highest PaO_2_/FIO_2_ ratio; strategy B (higher VT + lower PEEP) was associated with the highest pulmonary compliance; strategy A (lower VT + lower PEEP) was associated with a shorter length of ICU stay; and strategy D (lower VT + zero end-expiratory pressure (ZEEP)) was associated with the lowest PaO_2_/FiO_2_ ratio and pulmonary compliance.

**Conclusions:**

For ICU patients without ALI or ARDS, strategy C (lower VT + higher PEEP) was associated with the highest PaO_2_/FiO_2_ ratio. Strategy B (higher VT + lower PEEP) was superior to the other strategies in improving pulmonary compliance. Strategy A (lower VT + lower PEEP) was associated with a shorter length of ICU stay, whereas strategy D (lower VT + ZEEP) was associated with the lowest PaO_2_/FiO_2_ ratio and pulmonary compliance.

**Electronic supplementary material:**

The online version of this article (doi:10.1186/s13054-016-1396-0) contains supplementary material, which is available to authorized users.

## Background

It has been shown that the application of lung-protective mechanical ventilation with a low tidal volume can improve the prognosis of patients with acute lung injury (ALI) or acute respiratory distress syndrome (ARDS) [1–3]. Several clinical studies have attempted to optimize the ventilator management strategy to improve oxygenation and lung compliance, thereby reducing the length of intensive care unit (ICU) stay and the mortality of ICU patients without ALI or ARDS [4–6]. However, the optimal mechanical ventilation strategy for ICU patients without ALI or ARDS is uncertain.

Clinical data show that patients without a diagnosis of ALI or ARDS can benefit ventilation with a low tidal volume [4, 7]. Schultz et al. [7] concluded that the initial ventilator setting, high tidal volume, may be associated with lung injury in patients without ALI or ARDS. Serpa Neto et al. [8] used a traditional pairwise meta-analysis to systematically evaluate ventilation strategies such as high and low tidal volume. Their conclusions were as follows: compared to ventilation with a higher tidal volume, protective ventilation with lower tidal volumes at the onset of mechanical ventilation was associated with better clinical outcomes, including a shorter length of hospital stay, lower mortality, fewer pulmonary infections, and less atelectasis, among patients without ALI or ARDS.

In addition to tidal volume, there are other factors in the overall ventilation strategy, such as positive end-expiratory pressure (PEEP), recruitment maneuver (RM), and respiratory ratio. Tidal volume and PEEP play important roles. However, traditional pairwise meta-analysis can only be used to compare specific factors between ventilation strategies and cannot be used to compare the entire set of parameters relevant to different ventilation strategies. Therefore, tidal volume and clinical outcomes of different comprehensive ventilation strategies using a specific tidal volume and PEEP cannot be compared by traditional pairwise meta-analysis. Accordingly, the results obtained from traditional pairwise meta-analyses have significant limitations. Fortunately, a network meta-analysis is advantageous for the evaluation of the comparative effectiveness of multiple interventions, even when some parameters might not have been directly compared. Additionally, network meta-analysis has the potential to reduce the uncertainty in treatment effect estimates [9, 10]. Given these advantages, we used a network meta-analysis to search the literature for data examining the optimal tidal volume and PEEP in patients without ALI or ARDS. Based on these data, we divided the ventilation mode into six types and considered each ventilation mode as a unique ventilation strategy. Subsequently, the effectiveness and safety of various ventilation strategies were compared to identify the optimal ventilation strategy for ICU patients without ALI or ARDS.

## Methods

We conducted our systematic review in accordance with the methods recommended in the Preferred Reporting Items for Systematic Reviews and Meta-Analyses (PRISMA) guidelines [11].

### Literature search

The trials were identified through electronic and manual searches. We searched the Cochrane Central Register of Controlled Trials (CENTRAL) in the Cochrane Library, EMBASE, MEDLINE, CINAHL, and Web of Science using a combination of MeSH terms and text words. We did not restrict our search based on language or year of publication. The most recent search date was July 2015. We reviewed the reference lists of published meta-analyses. In addition, we manually searched the bibliographies of randomized controlled trials, meta-analyses, and systematic reviews for relevant studies that may have been missed in the initial electronic search.

### Inclusion and exclusion criteria

The study inclusion and exclusion process was conducted separately by two groups. When there was a discrepancy between the two groups, the selection committee met to reach a consensus on the inclusion or exclusion of the disputed article. We first excluded the following types of articles: reviews, retrospective studies, observational studies, case reports, animal studies, studies conducted on children, studies examining only psychological mechanisms, unrelated studies (such as studies of mechanical ventilation in patients with ARDS), duplicate reports, studies involving repeated experiments (commentary articles on specific studies or secondary analyses of experimental data), and nonrandomized trials. Ultimately, randomized controlled trials examining mechanical ventilation in ICU patients without ALI or ARDS were included. All of the included studies were of relatively high quality with a low risk of bias. No studies were excluded because of quality concerns.

### Outcome measures and data extraction

The extracted data included basic study information such as the experimental design, experimental period, country of the study, inclusion criteria, age and gender of the included patients, detailed experimental procedures, specific mechanical ventilation settings, clinical outcomes, and safety outcomes of the patients. The primary outcome of this study was the PaO_2_/FiO_2_ ratio. If multiple PaO_2_/FiO_2_ ratios were presented in a report, the last result was used. The secondary outcomes of this study were pulmonary compliance and the duration of ICU stay. Two groups extracted the data separately and then performed comparison and verification together. If necessary, we contacted the corresponding authors to seek assistance in the case of missing data and sent a table containing the extracted data to those authors for supplementary data or verification.

### Ventilation strategies

In this network meta-analysis, the parameters of ventilation strategies for ICU patients without ARDS were specified. Lower PEEP was defined as PEEP lower than 10 mmHg, and higher PEEP was defined as PEEP higher than or equal to 10 mmHg [12]. Lower tidal volume was defined as lower than or equal to 8 ml per kg predicted body weight, and higher tidal volume was defined higher than 8 ml per kg predicted body weight [13]. Accordingly, six ventilation strategies were obtained (Table 1).

**Table 1:** Six ventilation strategies for intensive care unit patients without ALI or ARDS

	Strategy
A	Lower tidal volume and lower PEEP (lower VT + lower PEEP)
B	Higher tidal volume and lower PEEP (higher VT + lower PEEP)
C	Lower tidal volume and higher PEEP (lower VT + higher PEEP)
D	Lower tidal volume (lower VT + ZEEP)
E	Higher tidal volume (higher VT+ ZEEP)
F	Higher tidal volume and higher PEEP (higher VT + higher PEEP)

Lower positive end-expiratory pressure (PEEP) <10 mmHg; higher PEEP ≥10 mmHg; lower tidal volume (VT) ≤8 ml/kg; higher VT >8 ml/kg. *ZEEP* zero end-expiratory pressure

### Statistical analysis

Network meta-analysis combines the direct and indirect evidence for all relative treatment effects and provides estimates with maximum power [14–16]. A network meta-analysis was performed using the GeMTC [17] package in R (i386 3.0.2). In this analysis, to maximize accuracy and power, the mean difference (MD) and 95 % confidence intervals were used to evaluate the effect of each mechanical ventilation strategy on pulmonary compliance and the PaO_2_/FiO_2_ ratio of ICU patients without ARDS or ALI [18]. A difference was considered statistically significant when the range of the 95 % confidence intervals did not include zero.

Model selection was based on the Dias guidelines [19] for evaluating linear models. Dbar denotes the posterior mean of the residual deviance; pD denotes the effective number of parameters (leverage); and DIC denotes the deviance information criterion. A smaller Dbar value indicates a better model fit. However, the model with the lowest DIC is generally chosen to aid the interpretation by accounting for model complexity. A lower DIC value indicates a better model fit. Differences between the models of less than 3 to 5 were not considered significant [20]. The models were run for 150,000 iterations, and convergence was assessed using the Brooks-Gelman-Rubin diagnostic approach [21]. We used a technique referred to as “back-calculation” [22] to evaluate the consistency of the findings of the network meta-analysis based on direct versus indirect evidence. During this process, three types of models were estimated: unrelated study effects, unrelated mean effects, and consistency.

The output of the summary function can be plotted for a visual representation. We investigated the possibility of statistical heterogeneity and inconsistency between the direct and indirect effect estimates by visually inspecting the forest plots and the *I*^2^ statistic using the Higgins–Thompson method (low heterogeneity 25 %, moderate 50 %, and high 75 %) [23]. We also ranked the different interventions in terms of their likelihood of leading to the best results for each outcome [12]. In the Markov chain Monte Carlo cycle, each ventilation strategy was ranked based on the estimated effect size. These probabilities summed to 1 for each treatment and each rank. *X*% means that the strategy achieves *x*% effectiveness. Thus, a higher percentage denotes a more effective intervention, although this ranking refers to only the considered possibilities rather than the actual effectiveness of a given ventilation strategy [18].

## Results

**Fig. 1:**
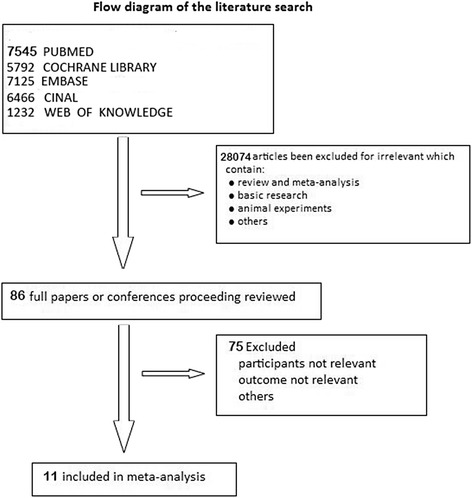
Flow diagram of the literature search

We identified 28,160 studies for review based on their titles and abstracts (Fig. 1). After an initial screen, we retrieved the full texts of 86 potentially eligible articles for a detailed assessment. Ultimately, we excluded 75 irrelevant full-text articles (Additional file 1), and 11 randomized controlled trials [4, 24–33] were included in the network meta-analysis. These studies included 575 patients who received one of six ventilation modes (Table 1). Unfortunately, ventilation strategies E (higher VT + ZEEP) and F (higher VT + higher PEEP) were isolated from the other ventilation strategies. Therefore, only the other four ventilation strategies were compared. All of the included studies were randomized controlled trials (Table 2).

**Table 2:** Characteristics of intensive care unit patients without acute lung injury or acute respiratory distress syndrom included in randomized controlled trials

Study	Country	Research period	Ventilation strategies	Patients (*n*)	Cause	Study quality assessment, Jadad scale	Results				
							PaO2/FIO2	Lung compliance (ml/cmH2O)	Deaths (*n*)	Length of ICUstay (days)	Length of hospital stay (days)
Lee PC [24] 1990	USA	10/1987-02/1988	A (lower VT + lower PEEP) vs B (higher VT + lower PEEP)	103	Multiple trauma or celiotomy	5	294 ± 86/260 ± 78	NR	NR	4.6 ± 1/2.7 ± 0.5	NR
Borges DL [25] 2013	Brazil	01/2011-03/2012	A (lower VT + lower PEEP) vs C (lower VT + higher PEEP)	89	CABG surgery	6	270 ± 90/328.25 ± 84.75	47.4 ± 12.5/55.8 ± 19.1	NR	NR	NR
Dyhr T [26] 2002	Denmark	NR	A (lower VT + lower PEEP) vs D (lower VT + ZEEP)	15	CABG surgery	7	379.5 ± 90/304.5 ± 97.5	58 ± 11/34 ± 10	NR	NR	NR
Chaney MA [27] 2000	America	NR	B (higher VT + lower PEEP) vs A (lower VT + lower PEEP)	25	CABG surgery	5	368.6 ± 93.6/395.1 ± 179.6	58 ± 11.4/48.2 ± 23	NR	NR	NR
Wrigge H [28] 2005	Germany	NR	B (higher VT + lower PEEP) vs C (lower VT + higher PEEP)	44	CABG surgery	6	NR	NR	NR	1.2 ± 0.5/ 2.1 ± 0.5	NR
Koutsoukou A [29] 2006	Greece	2005	D (lower VT+ ZEEP) vs A (lower VT + lower PEEP)	21	Severe brain damage	6	NR	NR	NR	NR	NR
Good JT Jr [30] 1979	America	NR	B (higher VT + lower PEEP) vs E (higher VT + ZEEP)	24	Open heart surgery	7	NR	NR	NR	NR	NR
Marvel SL [31] 1986	America	1983	E (higher VT + ZEEP) vs B (higher VT + lower PEEP) vs F (higher VT + higher PEEP)	44	CABG surgery	7	NR	NR	NR	NR	8.9 ± 0.4/8.8 ± 0.5
Zupancich E [32] 2005	Italy	NR	B (higher VT + lower PEEP) vs C (lower VT + higher PEEP)	40	CABG surgery	6	324 ± 120/344 ± 94	NR	NR	NR	NR
Pinheiro deOliveira R [33] 2010	Brazil	NR	B (higher VT + lower PEEP) vs A (lower VT + lower PEEP)	20	Surgery and trauma	6	334.25 ± 82.3/299.5 ± 71.9	NR	4/3	6.5 ± 5/7.7 ± 7.6	NR
Determann RM [4] 2010	Netherlands	01/2005-12/2007	E (higher VT + ZEEP) vs D (lower VT+ ZEEP)	150	Neurosurgery/neurology, cardiothoracic surgery, and cardiology	7	NR	NR	23/24	NR	NR

The ventilation strategies are described in “Table 1”. *PaO2/FIO2* partial pressure of arterial oxygen/fraction of inspired oxygen, *PEEP* positive end-expiratory pressure, *VT* tidal volume, *ZEEP* zero end-expiratory pressure, *NR* no result reported, *CABG* coronary artery bypass graft

### Heterogeneity

In this network meta-analysis, six studies reported data on the PaO_2_/FIO_2_ ratio and were included in the meta-analysis. These studies were two-arm trials. The comparison between these studies showed no heterogeneity (Additional file 2A). Among all of the included studies, pulmonary compliance was reported in three articles. These studies were two-arm trials, and the comparison between these studies did not show any heterogeneity (Additional file 2B).

### PaO_2_/FIO_2_ ratio

**Fig. 2:**
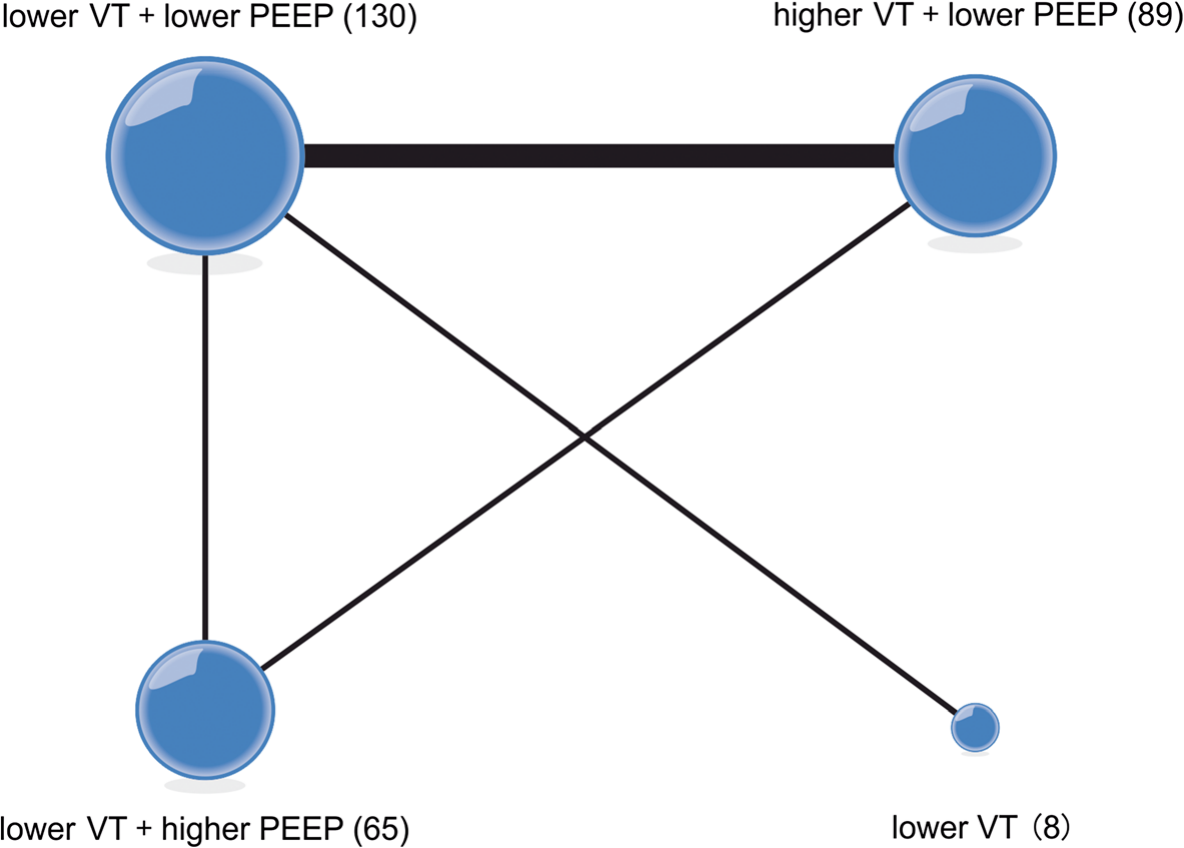
Network of the comparisons of the partial pressure of arterial oxygen/fraction of inspired oxygen ratio in the Bayesian network meta-analysis. The size of a given *node* is proportional to the number of patients (*in parentheses*) randomized to receive the treatment. The *width* of each *line* is proportional to the number of trials (specified next to the line) comparing the connected treatments. *PEEP* positive end-expiratory pressure, *VT* tidal volume

Six articles reported the PaO_2_/FIO_2_ ratio [24–27, 32, 33]. We chose a fixed-effects model (Additional file 3A) to evaluate the MDs in the overall effect sizes between the four compared ventilation strategies (Fig. 2). The PaO2/FIO2 ratios, MD values and 95 % confidence intervals of various ventilation strategies are shown in Additional file 4A.

pt?>For probability ranking: in the rankings of the compared ventilation strategies in terms of the PaO_2_/FIO_2_ ratio (Additional file 5A), we found that strategy C (lower VT + higher PEEP) had the greatest potential to improve the PaO_2_/FIO_2_ ratio; the probability of strategy C holding the top ranking was 98.8 %. Strategy D (lower VT + ZEEP) was estimated to be the worst strategy in terms of the PaO_2_/FIO_2_ ratio.

**Fig. 3:**
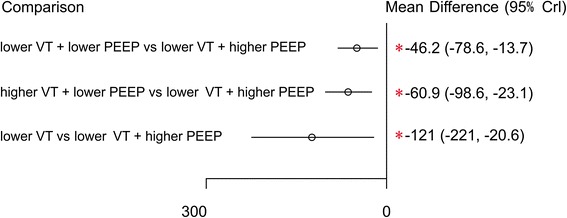
Mean difference in the partial pressure of arterial oxygen/fraction of inspired oxygen (PaO_2_/FIO_2_) ratio relative to the PaO_2_/FIO_2_ ratio of ventilation strategy C based on Bayesian network meta-analysis. *Crl* credible interval for Bayesian network meta-analysis. The mean difference (*MD*) was estimated from a Bayesian random-effects model of PaO_2_/FIO_2_ ratios in the network. ^*^The range of 95 % confidence intervals does not contain zero. MD <0 favors strategy C. *PEEP* positive end-expiratory pressure, *VT* tidal volume

For direct and indirect comparison: compared to strategies B (higher VT + lower PEEP), A (lower VT + lower PEEP), and D (lower VT + ZEEP), strategy C (lower VT + higher PEEP) had the greatest potential to improve the PaO_2_/FIO_2_ ratio; the respective MDs (95 % confidence intervals) were −46.2 (−78.6, −13.7), −60.9 (−98.6, −23.1), and −121 (−221, −20.6) (Fig. 3).

### Pulmonary compliance

**Fig. 4:**
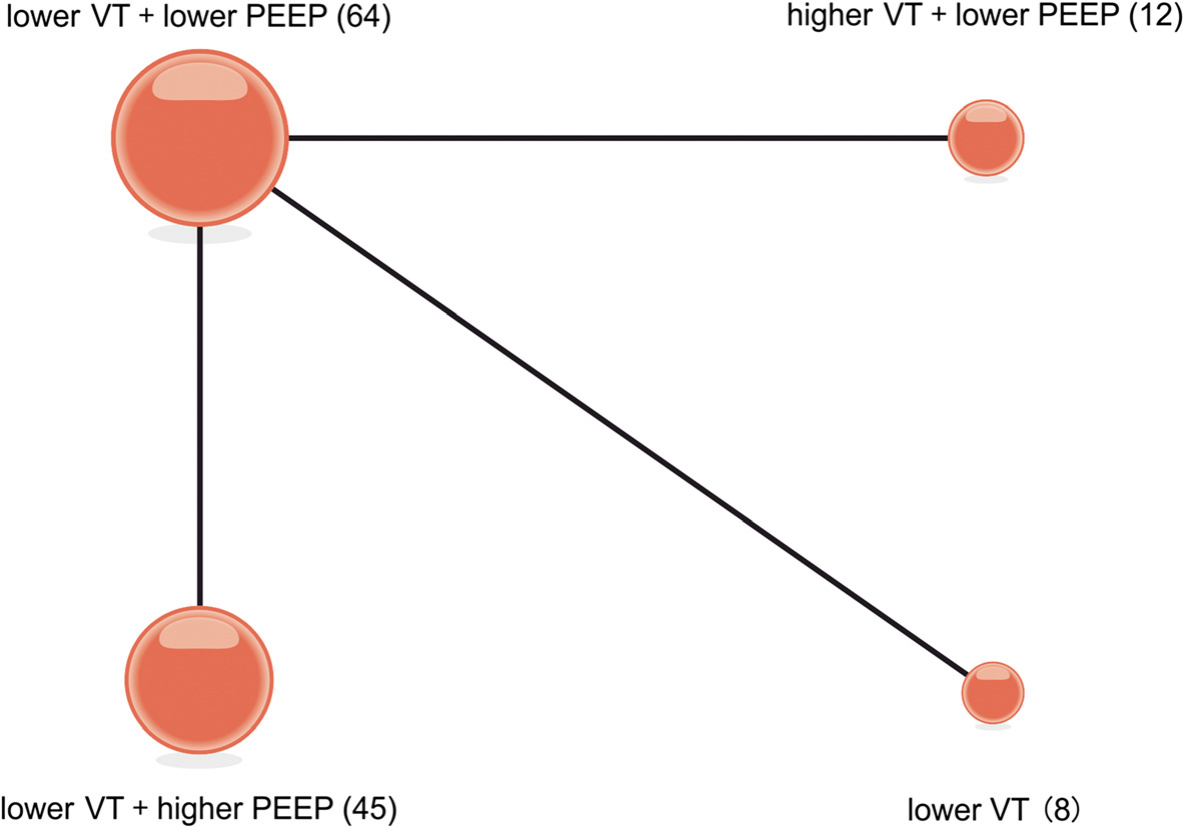
Network of the comparisons of pulmonary compliance in the Bayesian network meta-analysis. The size of a given *node* is proportional to the number of patients (*in parentheses*) randomized to receive the treatment. The *width* of each *line* is proportional to the number of trials (specified next to the line) comparing the connected treatments. *PEEP* positive end-expiratory pressure, *VT* tidal volume

Three articles [25–27] reported on pulmonary compliance and examined four ventilation strategies (Fig. 4). We chose a fixed-effects model (Additional file 3B) to evaluate the MDs in the overall effect sizes. MD values and 95% confidence intervals of various ventilation strategies are shown in Additional file 4B.

For probability ranking: we summarized the rankings of the compared ventilation strategies in terms of pulmonary compliance (Additional file 5B). Ventilation strategy B (higher VT + lower PEEP) had the greatest potential to improve pulmonary compliance; the probability of strategy B holding the top ranking was 57.2 %, followed by strategy C (lower VT + higher PEEP) at 42.7 %. Strategy D (lower VT + ZEEP) was estimated to be the worst strategy in terms of pulmonary compliance.

**Fig. 5:**
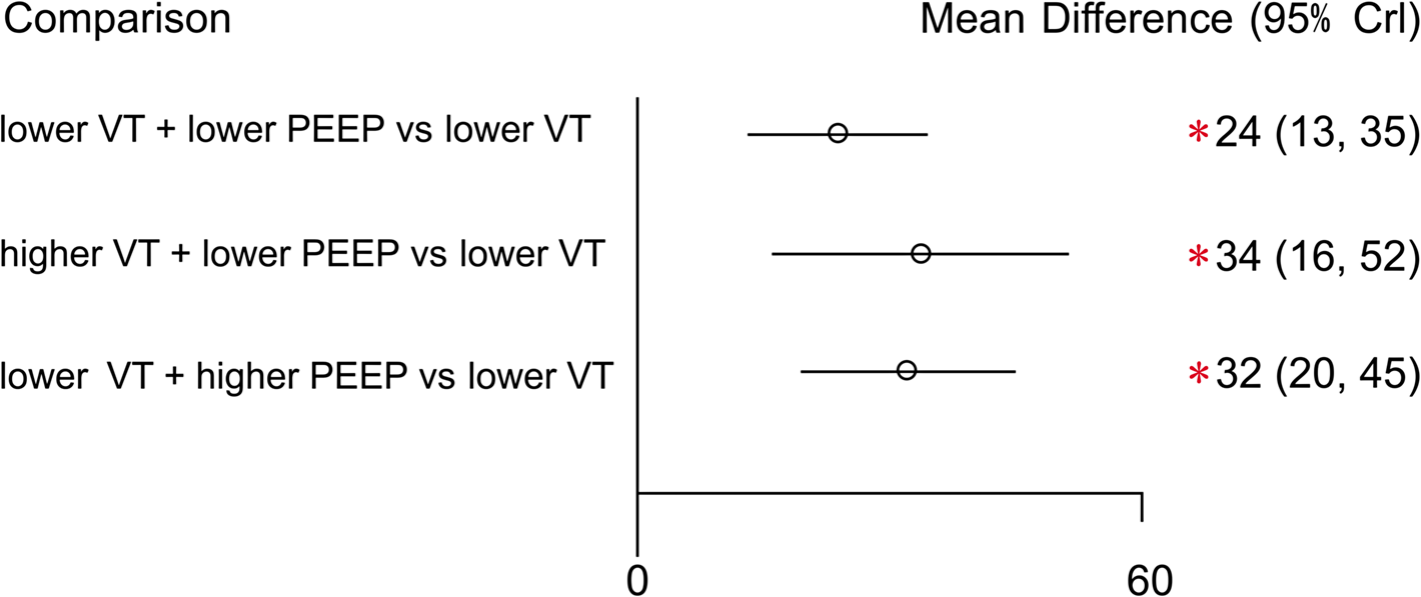
Mean deviance in pulmonary compliance relative to strategy D based on Bayesian network meta-analysis. *Crl* credible interval for Bayesian network meta-analysis. The mean difference (*MD*) was estimated from a Bayesian random-effects model of the pulmonary compliances in the network. ^*^The range of 95 % confidence intervals does not contain zero. MD >0 favors strategies A, B and C. *PEEP* positive end-expiratory pressure, *VT* tidal volume

For direct and indirect comparison: compared to ventilation strategy D (lower VT+ ZEEP), strategies A (lower VT + lower PEEP), B (higher VT + lower PEEP), and C (lower VT + higher PEEP) were associated with an improvement in lung compliance; the respective MDs (95 % confidence intervals) were 24 (13, 25), 34 (16, 52), and 32 (20, 45) (Fig. 5).

### Length of ICU stay

**Fig. 6:**
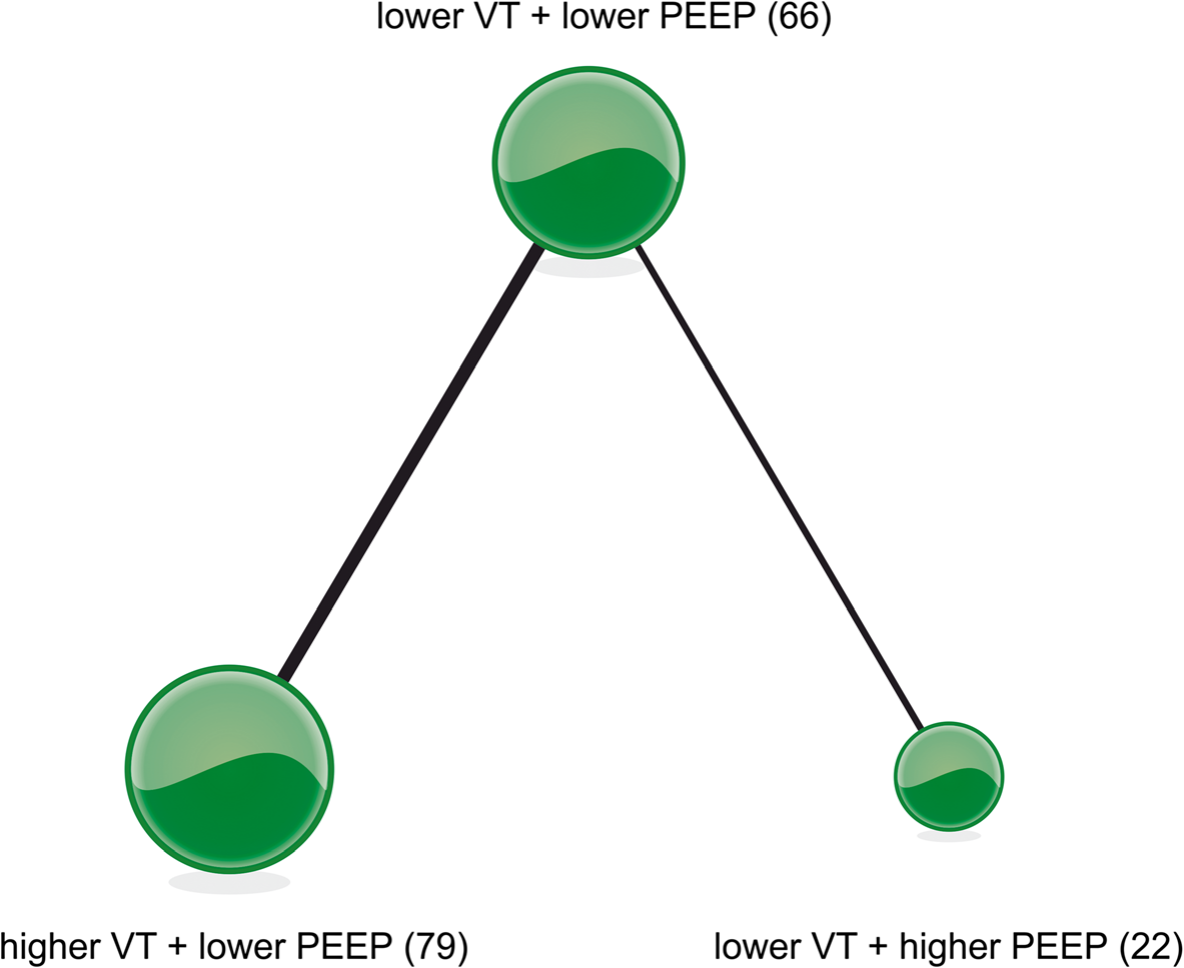
Network of the comparisons of the length of ICU stay in the Bayesian network meta-analysis. The size of a given *node* is proportional to the number of patients (*in parentheses*) randomized to receive the treatment. The *width* of each *line* is proportional to the number of trials (specified next to the line) comparing the connected treatments. *PEEP* positive end-expiratory pressure, *VT* tidal volume

Three articles reported on the secondary outcome of the length of ICU stay [24, 28, 33], and these studies examined three ventilation strategies (Fig. 6). We chose a fixed-effects model (Additional file 3C) to evaluate the MDs in the overall effect sizes. MD values and 95% confidence intervals of various ventilation strategies are shown in Additional file 4B.

For probability ranking: strategy A (lower VT + lower PEEP) was associated with a shorter length of ICU stay; and the probability of strategy A holding the top ranking was 98.7 %. Strategy B (higher VT + lower PEEP) was estimated to be the worst strategy in terms of the length of ICU stay (Additional file 5C).

**Fig. 7:**
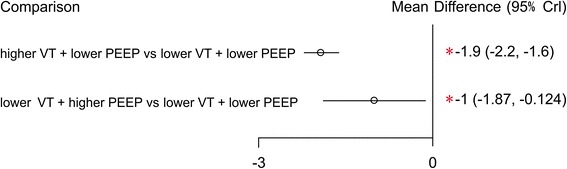
Mean difference in the length of ICU stay relative to strategy A based on Bayesian network meta-analysis. *CI* credible interval for Bayesian network meta-analysis. The mean difference (*MD*) was estimated from a Bayesian random-effects model of the lengths of ICU stay in the network. ^*^The range of 95 % confidence intervals does not contain zero. MD <0 favors strategy A. *PEEP* positive end-expiratory pressure, *VT* tidal volume

For direct and indirect comparison: compared to ventilation strategies B (higher VT + lower PEEP) and C (lower VT + higher PEEP), strategy A (lower VT + lower PEEP) was associated with a shorter length of ICU stay, and the respective MDs (95 % confidence intervals) were −1.9 (−2.2, −1.6) and −1 (−1.87, −0.124) (Fig. 7).

### Other outcomes

Only one study [31] reported on the length of hospital stay, and two studies [4, 33] reported on the number of deaths. Unfortunately, certain ventilation strategies were isolated from the remaining ventilation strategies in these studies; therefore, the aforementioned outcomes could not be examined via network meta-analysis.

## Discussion

Serpa Neto et al. [8] published a traditional pairwise meta-analysis on ventilation strategies for patients without ALI or ARDS in 2012; this meta-analysis applied no restrictions on the setting (ICU or operating room). However, considering the effect of the surgical procedure, ventilation during an operation and ventilation in the ICU are different, and this difference could lead to heterogeneous results. Therefore, we included only randomized controlled trials of ICU patients without ALI or ARDS. This approach renders this meta-analysis more purposeful and scientific.

However, network meta-analysis has several shortcomings. It is difficult to understand its methodological aspects. This method is not perfect and poses various challenges; for instance, we should carefully assess both conceptual and statistical heterogeneity as well as incoherence between included studies [34]. Furthermore, the results of a network meta-analysis are presented in two ways: probability ranking and the results of combined direct and indirect comparisons. The estimates of treatment effects should be interpreted with caution due to their uncertainty because treatment rankings or probabilities can be misleading [34], whereas a combined analysis of direct and indirect evidence produces more meaningful results based on published studies.

In this study, the PaO_2_/FIO_2_ ratio was the primary outcome. Probability ranking showed that ventilation strategy C (lower VT + higher PEEP) was associated with the greatest increase in oxygenation; in contrast, ventilation strategy D (lower VT+ ZEEP) ranked last and was associated with the lowest PaO_2_/FIO_2_ ratio among all of the ventilation strategies examined. Moreover, based on direct and indirect comparisons, compared to strategies A (lower VT + lower PEEP), B (higher VT + lower PEEP), and D (lower VT + ZEEP), ventilation strategy C (lower VT + higher PEEP) was the most effective ventilation strategy in terms of the PaO_2_/FiO_2_ ratio, and these differences in effectiveness were statistically significant.

Ventilator-associated lung injury is a common clinical complication in critically ill patients receiving mechanical ventilation [35]. An increased tidal volume can overstretch the alveoli; this process is termed volutrauma, and overstretching is the main reason for ventilator-associated lung injury [35, 36]. Although lower tidal volumes can cause distal alveolar collapse and inadequate ventilation, high PEEP can significantly compensate for this shortcoming by stimulating recruitment of collapsed alveoli to alleviate focal atelectasis, increase alveolar ventilation, and reduce the alveolar-arterial oxygen difference, thereby effectively relieving the occurrence of pulmonary shunting and ensure the delivery of arterial oxygen [37, 38]. Simultaneously, a reduced tidal volume can reduce ventilator-associated lung injury [37–39]. These phenomena could explain how ventilation strategy C (lower VT + higher PEEP) increased the PaO_2_/FiO_2_ ratio and restored oxygen saturation.

Pulmonary compliance was a secondary outcome in our study. Probability ranking indicated that ventilation strategy B (higher VT + lower PEEP) was associated with the highest pulmonary compliance but that ventilation strategy D (lower VT+ ZEEP) was associated with the lowest pulmonary compliance. Moreover, based on direct and indirect comparisons, we found that compared to strategies A (lower VT + lower PEEP), B (higher VT + lower PEEP), and C (lower VT + higher PEEP), strategy D (lower VT + ZEEP) was significantly associated with the lowest lung compliance.

The finding that ventilation strategy B (higher VT + lower PEEP) was associated with the highest pulmonary compliance could be related to the following reasons: (1) a high tidal volume during mechanical ventilation can expand small airways, fully open alveoli, and reduce intraoperative focal atelectasis, and PEEP can further increase the stability of opened alveoli and enhance pulmonary compliance [40]; (2) PEEP can hold alveoli open at the end of exhalation, significantly increase functional residual capacity, enable the alveoli to begin to expand at a high functional residual capacity, avoid excessive expansion and contraction of the lungs during inhalation and exhalation, and reduce the destruction of lung tissue and its interstitial structure, and damage to the alveoli. Consequently, PEEP maintains the elastic recoil of the lung and enhances lung compliance [38, 40, 41].

Ventilation strategy D (lower VT + ZEEP) was associated with the lowest pulmonary compliance and the smallest increase in oxygenation. The mechanism underlying this association could be that a lower tidal volume during mechanical ventilation causes the distal alveolar and small airways to close, resulting in alveolar collapse, insufficient ventilation, increased intrapulmonary shunting, and a decreased PaO_2_/FIO_2_ ratio. This mechanism is supported by the findings of HU et al. [42]. Hedenstierna et al. [43] also found that atelectasis and airway closure can explain 75 % of the deterioration in PaO_2_ during mechanical ventilation. Under conditions of alveolar collapse and reduced functional residual capacity resulting from a low tidal volume, lung compliance cannot be enhanced effectively. This result is consistent with the results presented by Bruno Enekvist: low pulmonary compliance may be correlated with an increased number of collapsed alveoli [44, 45].

There were three reports on the duration of ICU stay in our network meta-analysis, and those reports examined ventilation strategies A (lower VT + lower PEEP), B (higher VT + lower PEEP) and C (lower VT + higher PEEP). Compared to strategies B (higher VT + lower PEEP) and C (lower VT + higher PEEP), strategy A (lower VT + lower PEEP) was significantly associated with a shorter duration of ICU stay. One reason for this benefit of strategy A is that lower tidal volume combined with lower PEEP can leave areas of alveolar collapse unaltered, avoiding cyclic recruitment/de-recruitment of distal lung units, while avoiding hyperinflation in normal lung regions, thus reducing end-inspiratory stress and lung inflammation and consequently minimizing ventilator-induced lung injury [46–48]. In addition, the use of a low tidal volume together with lower PEEP (5 cm H_2_0) in animal models can result in improved oxygenation [49]. This finding is consistent with the results that were described by Karsten et al. [50], who stated that lower PEEP combined with low VT prevents deoxygenation when there is pneumoperitoneum and leads to a lower atelectasis score based on computed tomography up to 2 hours postoperatively.

This study had several limitations. The initial aim of this article was focused on the development of lung injury, overall survival, the incidence of pulmonary infection and atelectasis, the length of ICU and hospital stay, time to extubation, the PaO_2_/FIO_2_ ratio, and pulmonary compliance; however, because of the small number of research articles that we included, there were no uniform outcome measures in our study. Aside from PaO_2_/FIO_2_ ratio and pulmonary compliance, we extracted only one article on hospital length of stay and only two articles on overall survival. Moreover, the original results were incomplete, which indicates that this meta-analysis could only produce relatively simple results rather than comprehensive and diverse results. We hope that there will be additional clinical research focused on the development of lung injury in the future.

## Conclusion

The results of this meta-analysis showed that for ICU patients without ALI or ARDS, strategy C (lower VT + higher PEEP) was associated with the highest PaO_2_/FiO_2_ ratio; strategy B (higher VT + lower PEEP) was superior to the other strategies in improving pulmonary compliance; strategy A (lower VT + lower PEEP) was associated with a shorter length of ICU stay; and strategy D (lower VT + ZEEP) was associated with the lowest PaO_2_/FiO_2_ ratio and pulmonary compliance.

## Key messages

- Strategy C (lower VT + higher PEEP) was associated with the highest PaO_2_/FiO_2_ ratio in ICU patients without ALI or ARDS
- Strategy B (higher VT + lower PEEP) was superior to the other strategies in improving pulmonary compliance in ICU patients without ALI or ARDS
- Strategy D (lower VT + ZEEP) was associated with the lowest PaO_2_/FiO_2_ ratio and pulmonary compliance in ICU patients without ALI or ARDS

## Abbreviations

ALI, acute lung injury; ARDS, acute respiratory distress syndrome; DIC, deviance information criterion; ICU, intensive care unit; MD, mean difference; PaO_2_/FiO_2_ ratio, partial pressure of arterial oxygen/fraction of inspired oxygen; PEEP, positive end-expiratory pressure; RM, recruitment maneuver; VT, tidal volume; ZEEP, zero end-expiratory pressure

## Additional files

Additional file 1: Appendix 1. Summary of excluded articles. (DOC 19 kb) Additional file 2: Appendix 2A. The PaO_2_/FIO_2_ ratio effect estimates from multiple treatment meta-analysis compared with direct and indirect estimates, based on back-calculated, and pairwise meta-analyses. Direct and indirect estimates of effect and the corresponding Bayesian *I* ^2^ values for inconsistency were calculated. The *I* ^2^ values from pooled pairwise meta-analysis for heterogeneity were also calculated. **Appendix 2B.** The ICU length of hospital effect estimates from multiple treatment meta-analysis compared with direct and indirect estimates, based on back-calculated, and pairwise meta-analyses. Direct and indirect estimates of effect and the corresponding Bayesian *I* ^2^ values for inconsistency were calculated. The *I* ^2^ values from pooled pairwise meta-analysis for heterogeneity were also calculated. (DOC 80 kb) Additional file 3: Appendix 3A. Model fit for PaO_2_/FIO_2_ ratio results. **Appendix 3B.** Model fit for compliance results. **Appendix 3C.** Model fit for ICU length of hospital stay results. (DOC 27 kb) Additional file 4: Appendix 4A. Pooled odds ratios for PaO2/FIO2 ratio. **Appendix 4B.** Pooled odds ratios for ICU length of hospital stay and pooled mean difference for pulmonary compliance. (DOC 32 kb) Additional file 5: Appendix 5A. Rankings based on simulations in terms of PaO2/FIO2 ratio. **Appendix 5B.** Rankings based on simulations in terms of compliance. **Appendix 5C.** Rankings based on simulations in terms of ICU length of hospital stay. (DOC 28 kb)
