# Supplementary material for: Mechanical ventilation strategies for intensive care unit patients without acute lung injury or acute respiratory distress syndrome: a systematic review and network meta-analysis
Source: Crit Care. 2016 Jul 22;20:226. doi: 10.1186/s13054-016-1396-0 (PMC4957383; doi:10.1186/s13054-016-1396-0)
Supplement: Additional file 1: Appendix 1. — Summary of excluded articles. (DOC 19 kb) [file 13054_2016_1396_MOESM1_ESM.doc]

**Appendix 1. Summary of excluded articles**

| **Year** | **Authors** | **Exclued Reasons** |
| --- | --- | --- |
| 1983 | GrazianoC | ventilation strategies do not match |
| 1990 | G.Conti | ventilation strategies do not match |
| 1994 | Sevenn H.Rappaport | ventilation strategies do not match |
| 1994 | S. Nava | patients do not match |
| 2002 | P. Reper | ventilation strategies do not match |
| 2004 | Hermann Wrigge | no relevant outcome |
| 2010 | [Oğurlu M](http://www.ncbi.nlm.nih.gov/pubmed?term=Oğurlu M%5BAuthor%5D&cauthor=true&cauthor_uid=20303833) | patients do not match |
| 2010 | Faizan Ahmed | ventilation strategies do not match |
| 2011 | Frederique Paulus | ventilation strategies do not match |
| 2011 | Sugantha Sundar | no relevant outcome |
| 2013 | Leme A | ventilation strategies do not match |
