# Supplementary material for: Mechanical ventilation strategies for intensive care unit patients without acute lung injury or acute respiratory distress syndrome: a systematic review and network meta-analysis
Source: Crit Care. 2016 Jul 22;20:226. doi: 10.1186/s13054-016-1396-0 (PMC4957383; doi:10.1186/s13054-016-1396-0)

**Appendix 2-A.** The PaO_2_/FIO_2_ ratio effect estimates from multiple treatment meta-analysis compared with direct and indirect estimates, based on back-calculated, and pair-wise meta-analyses. Direct and indirect estimates of effect and the corresponding Bayesian ‘I^2^’ for inconsistency were calculated. And the‘I^2^’from Pooled pair-wise meta-analysis for heterogeneity were also calculated.


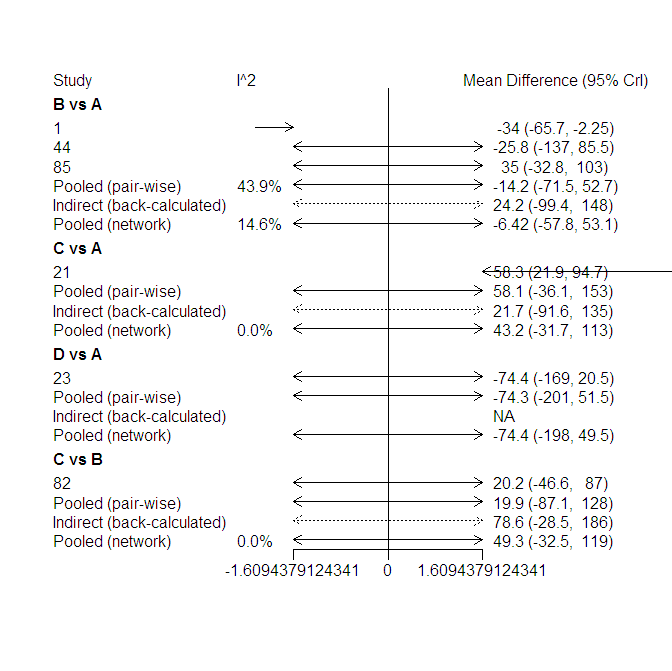


**Appendix 2-B.** The ICU length of hospital effect estimates from multiple treatment meta-analysis compared with direct and indirect estimates, based on back-calculated, and pair-wise meta-analyses. Direct and indirect estimates of effect and the corresponding Bayesian ‘I^2^’ for inconsistency were calculated. And the‘I^2^’from Pooled pair-wise meta-analysis for heterogeneity were also calculated.


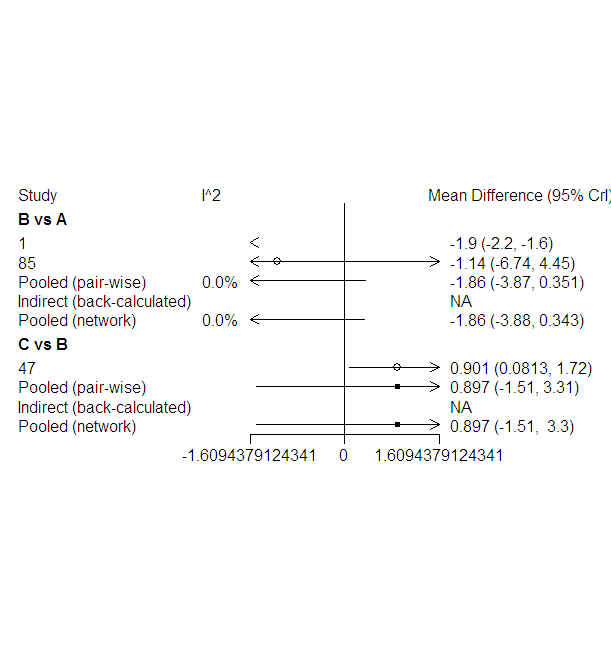

Supplement: Additional file 2: Appendix 2A. — The PaO2/FIO2 ratio effect estimates from multiple treatment meta-analysis compared with direct and indirect estimates, based on back-calculated, and pairwise meta-analyses. Direct and indirect estimates of effect and the corresponding Bayesian I 2 values for inconsistency were calculated. The I 2 values from pooled pairwise meta-analysis for heterogeneity were also calculated. Appendix 2B. The ICU length of hospital effect estimates from multiple treatment meta-analysis compared with direct and indirect estimates, based on back-calculated, and pairwise meta-analyses. Direct and indirect estimates of effect and the corresponding Bayesian I 2 values for inconsistency were calculated. The I 2 values from pooled pairwise meta-analysis for heterogeneity were also calculated. (DOC 80 kb) [file 13054_2016_1396_MOESM2_ESM.doc]
