# Supplementary material for: Mechanical ventilation strategies for intensive care unit patients without acute lung injury or acute respiratory distress syndrome: a systematic review and network meta-analysis
Source: Crit Care. 2016 Jul 22;20:226. doi: 10.1186/s13054-016-1396-0 (PMC4957383; doi:10.1186/s13054-016-1396-0)
Supplement: Additional file 3: Appendix 3A. — Model fit for PaO2/FIO2 ratio results. Appendix 3B. Model fit for compliance results. Appendix 3C. Model fit for ICU length of hospital stay results. (DOC 27 kb) [file 13054_2016_1396_MOESM3_ESM.doc]

**Appendix 3-A.Model fit for PaO_2_/FIO_2_ ratio– results**

|  | **Mean deviance** | **Penalty (pD)** | **DIC** |
| --- | --- | --- | --- |
| **Fixed effects model** | **110.443388** | **9.000791** | **119.444179** |
|
| **Random effects model** | **107.85513** | 1**0.75737** | **118.61250** |
|

Mean deviance indicates the posterior mean of the residual deviance. pD indicates the effective number of parameters (leverage).DIC indicates the ’Deviance Information Criterion’. A lower pD and DIC indicates a better model fit, based on the above information, fixed-effect model is the preferred model.

**Appendix 3-B. Model fit for compliance – results**

|  | **Mean deviance** | **Penalty (pD)** | **DIC** |
| --- | --- | --- | --- |
| **Fixed effects model** | **31.853780** | **6.012185** | **37.865965** |
|
| **Random effects model** | **31.84598** | **5.99531** | **37.84129** |
|

Mean deviance indicates the posterior mean of the residual deviance. pD indicates the effective number of parameters (leverage).DIC indicates the ’Deviance Information Criterion’. A lower pD and DIC indicates a better model fit, based on the above information, fixed-effect model is the preferred model

**Appendix 3-C. Model fit for ICU length of hospital – results**

|  | **Mean deviance** | **Penalty (pD)** | **DIC** |
| --- | --- | --- | --- |
| **Fixed effects model** | **3.207004** | **4.997863** | **8.204867** |
|
| **Random effects model** | **3.371305** | **5.183164** | **8.554470** |
|

Mean deviance indicates the posterior mean of the residual deviance. pD indicates the effective number of parameters (leverage).DIC indicates the ’Deviance Information Criterion’. A lower pD and DIC indicates a better model fit, based on the above information, fixed-effect model is the preferred model.
