# Supplementary material for: Mechanical ventilation strategies for intensive care unit patients without acute lung injury or acute respiratory distress syndrome: a systematic review and network meta-analysis
Source: Crit Care. 2016 Jul 22;20:226. doi: 10.1186/s13054-016-1396-0 (PMC4957383; doi:10.1186/s13054-016-1396-0)
Supplement: Additional file 4: Appendix 4A. — Pooled odds ratios for PaO2/FIO2 ratio. Appendix 4B. Pooled odds ratios for ICU length of hospital stay and pooled mean difference for pulmonary compliance. (DOC 32 kb) [file 13054_2016_1396_MOESM4_ESM.doc]

**Appendix 4-A. Pooled odds ratios for PaO2/FIO2 ratio.**

| **A** | **-14.7**  (-40.9，11.4) | **46.2**  (13.7，78.6) | **-74.7**  (-170, 20.2) |
| --- | --- | --- | --- |
| .. | **B** | **60.9**  (23.1,98.6) | **-60**  (-159,38.6) |
| .. | .. | **C** | **-121**  (-221,-20.6.) |
| .. | .. | .. | **D** |

Mean difference for PaO2/FIO2 ratio are above the diagonal line (row defining treatment *vs* column defining treatment ). If the range of 95% CI for MD does not contain 0, the red numbers indicate corresponding values. ..=not compared. CI=credible interval. MD= mean difference.

**Appendix 4-B. Pooled odds ratios for ICU length of hospital and pooled mean difference for pulmonary compliance.**

| **A** | **9.9**  (-4.3, 24) | **8.4**  (1.7，15) | **-24**  (-35, -13) |
| --- | --- | --- | --- |
| -**1.9**  (-2.2, -1.6) | **B** | **1.5**  (-17, 14) | **-34**  (-52, -16) |
| **-1**  (-1.9,-0.1) | **0.9**  (-0.1., 1.7) | **C** | **-32**  (-45, -20) |
|  |  |  | **D** |

Mean difference for ICU length of hospital and pooled mean difference for compliance are above the diagonal line (row defining treatment *vs* column defining treatment ). If the range of 95% CI for MD does not contain 0, the red numbers indicate corresponding values. ..=not compared. CI=credible interval. MD= mean difference.
