# Supplementary material for: Mechanical ventilation strategies for intensive care unit patients without acute lung injury or acute respiratory distress syndrome: a systematic review and network meta-analysis
Source: Crit Care. 2016 Jul 22;20:226. doi: 10.1186/s13054-016-1396-0 (PMC4957383; doi:10.1186/s13054-016-1396-0)
Supplement: Additional file 5: Appendix 5A. — Rankings based on simulations in terms of PaO2/FIO2 ratio. Appendix 5B. Rankings based on simulations in terms of compliance. Appendix 5C. Rankings based on simulations in terms of ICU length of hospital stay. (DOC 28 kb) [file 13054_2016_1396_MOESM5_ESM.doc]

**Appendix 5-A. Rankings based on simulations in terms of PaO2/FIO2 ratio.**

| Rank | A | B | C | D |
| --- | --- | --- | --- | --- |
| 1 | 0.0024683333 | 0.0006583333 | **0.9877466667** | 0.0091266667 |
| 2 | **0.81075333** | 0.12726667 | 0.01178167 | 0.05019833 |
| 3 | 0.17868833 | **0.76229333** | 0.00045500 | 0.05856333 |
| 4 | 8.090000e-03 | 1.097817e-01 | 1.666667e-05 | **8.821117e-01** |

The numbers in the table represent the probability that each treatment is best (rank 1), the second highest (rank 2), etc. The numbers in bold text represent the highest likelihood of ranking for each particular treatment. Higher rank indicates higher PaO2/FIO2 ratio.

**Appendix 5-B. Rankings based on simulations in terms of compliance**.

| Rank | A | B | | C | D |
| --- | --- | --- | --- | --- | --- |
| 1 | 0.0006366667 | | **0.5725350000** | 0.4268283333 | 0.0000000000 |
| 2 | 9.110000e-02 | 3.422217e-01 | | **5.666767e-01** | 1.666667e-06 |
| 3 | **9.082533e-01** | 8.516333e-02 | | 6.495000e-03 | 8.833333e-05 |
| 4 | 0.00001 | 0.00008 | | 0.00000 | **0.99991** |

The numbers in the table represent the probability that each treatment is worst (rank 1), the second worst (rank 2), etc. The numbers in bold text represent the highest likelihood of ranking for each particular treatment. Higher rank indicates higher incidence of compliance.

**Appendix 5-C. Rankings based on simulations in terms of ICU length of hospital.**

| Rank | A | B | C |
| --- | --- | --- | --- |
| 1 | **0.98731167** | 0.00000000 | 0.01268833 |
| 2 | 0.01268833 | 0.01583167 | **0.97148000** |
| 3 | 0.00000000 | **0.98416833** | 0.01583167 |

The numbers in the table represent the probability that each treatment is worst (rank 1), the second worst (rank 2), etc. The numbers in bold text represent the highest likelihood of ranking for each particular treatment. Higher rank indicates higher incidence of ICU length of hospital.
